# Supplementary material for: Kin discrimination allows plants to modify investment towards pollinator attraction
Source: Nat Commun. 2018 May 22;9:2018. doi: 10.1038/s41467-018-04378-3 (PMC5964244; doi:10.1038/s41467-018-04378-3)
Supplement: Supplementary file 1 — Supplementary Information [file 41467_2018_4378_MOESM1_ESM.pdf]

Supplementary Information

Kin discrimination allows plants to modify investment towards pollinator attraction

Torices et al.

Nature Communications

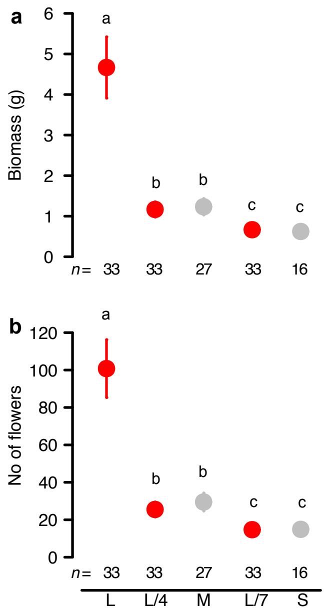

Supplementary Figure 1 | Effect of soil volume and pot size on plant size and floral display of solitary plants. Least-squared means (± s.e.m.) of above-ground plant biomass (a), and number of flowers (b) of plants grown in large pots (L) with 1.5 l of soil, in medium-sized pots (M) with 0.38 l of soil, and in small pots (S) with 0.21 l of soil. The value of plants in large pots but divided by 4 (L/4) and by 7 (L/7) was included for a direct comparison with M and S pots, respectively. Different letters indicate significant differences between treatments (GLMM, $P < 0.05$, after Holm's adjustment). Sample sizes are indicated below each symbol.

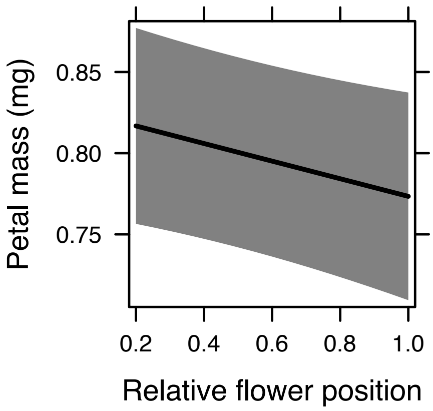

Supplementary Figure 2 | Flower petal mass as a function of its position in the inflorescence. Results of the linear mixed model are shown, with petal mass as the response variable and the relative flower position as the explanatory variable. The grey area indicates the 95% confidence interval. Individual plant was included as random factor, nested in plant family. Relative flower position indicates the position of the flower within the inflorescence from bottom to top. The index is bound between 0 and 1, representing the lowest position and highest flowers in the inflorescence, respectively. Petal mass declined towards the inflorescence apex (n = 1151, $F_{1, 879} = 4.43$, $P = 0.035$).

Supplementary Table 1 | Effects of resource limitation caused by either plant competition or by soil resource deprivation.

	3 neighbour groups			6 neighbour groups		
	n	χ^2	*P*	n	χ^2	*P*
Advertising effort	117	8.62	0.035	94	30.05	<0.001
Number of flowers	118	91.21	<0.001	95	101.75	<0.001
Mean petal mass	119(310)	9.62	0.022	95(242)	18.74	<0.001
Individual biomass	117	140.12	<0.001	95	174.65	<0.001
Height	118	31.62	<0.001	95	16.23	0.001

Generalized linear mixed models were fitted independently for each response variable, and for each group size, to compare plants grown with kin and/or with non-kin with those grown alone (Figs 1 and 2, Supplementary Table 2). We included two types of solitary plants with two levels of soil volume: large pots, where solitary plants had the same amount of soil as pots with neighbours; and smaller pots, where solitary plants had the same soil volume as the proportion of soil that focal plants with neighbours had in their pots. Type-II tests of the main effect of experimental treatment on each response variable are shown. The relative position of the flower in the inflorescence was included as a covariate for models for mean petal mass. Plant family was included as random factor in all models, whereas individual plant was nested in plant family for the model for petal-mass variation. Plant biomass was log-transformed. Sample sizes (n) indicate the number of individuals and, for mean petal-mass, the number of flowers (in parentheses).

Supplementary Table 2 | Comparisons between floral display and plant size of solitary plants *vs*. focal plants grown within a group.

Solitary plant	*vs.*	Focal plant with *n* neighbours	Neighbours	
			Kin	Non-kin
Advertising effort				
In large pot		3	0.129	1.000
		6	<0.001	0.249
In medium pot		3	0.044	0.928
In small pot		6	<0.001	0.099
Flower number				
In large pot		3	<0.001	<0.001
		6	<0.001	<0.001
In medium pot		3	<0.001	0.043
In small pot		6	<0.001	<0.001
Mean petal mass				
In large pot		3	0.553	0.993
		6	0.132	0.420
In medium pot		3	0.022	0.148
In small pot		6	<0.001	0.145
Biomass				
In large pot		3	< 0.001	< 0.001
		6	< 0.001	< 0.001
In medium pot		3	< 0.001	0.034
In small pot		6	0.002	< 0.001
Height				
In large pot		3	0.063	0.063
		6	0.728	1.000
In medium pot		3	0.006	0.012
In small pot		6	0.038	0.008

p-value of the least-square mean comparison between solitary plants and focal plants, with three and six kin or non-kin neighbours (Supplementary Table 1). All *p*-values were corrected for multiple comparisons using Holm's adjustment.

Supplementary Table 3 | The effect of soil volume on the floral display and plant size of solitary plants.

	n	d.f.	χ^2	*P*
Advertising effort	76	2	1.35	0.508
Number of flowers	77	2	123.16	<0.001
Mean petal mass	78(200)	2	14.40	<0.001
Individual biomass	76	2	190.98	<0.001
Height	77	2	31.02	<0.001

Generalized linear mixed models were fitted independently for each response variable to compare the three types of pots: large pots with 1.5 l of soil, medium-sized pots with 1/4 of the soil of large pots, and small pots with 1/7 of the soil of large pots (Fig. 1). Type-II tests of the main effect of soil volume on each response variable are shown. The relative position of the flower in the inflorescence was included as a covariate for the petal-mass model. Plant family was included as a random factor in all models, whereas individual plant was nested in plant family for the model fitting variation in petal mass. Plant biomass was log-transformed. Sample sizes (n) indicate the number of individuals and, for mean petal-mass, the number of flowers (in parenthesis).
